# From Weakness to Wellness: A Rare Case of Severe Pancytopenia and Vitamin B12 Deficiency

**DOI:** 10.7759/cureus.44017

**Published:** 2023-08-24

**Authors:** Aagna Patel, Francis Chabot, Osman Hamid

**Affiliations:** 1 Department of Medicine, Lake Erie College of Osteopathic Medicine, Erie, USA; 2 Department of Family Medicine, Mohawk Valley Health System, Utica, USA

**Keywords:** cyanocobalamin, leukemia, macrocytic anemia, pancytopenia, vitamin b12 deficiency

## Abstract

Vitamin B12 (cobalamin) deficiency is a commonly seen nutritional deficiency that presents with a broad spectrum of clinical symptoms. In this report, we describe a case of a 49-year-old female patient who presented to the emergency department with sudden onset of a syncopal-like episode, generalized weakness, and severe pancytopenia, who was subsequently diagnosed with vitamin B12 deficiency upon admission. The patient underwent a thorough evaluation to exclude alternative etiologies for her presentation. Her clinical symptoms and blood count significantly improved after six days of treatment with vitamin B12 supplementation. While vitamin B12 deficiency is a commonly recognized issue, healthcare providers should be aware of its infrequent presentations. Our case serves as a reminder to clinicians to remain vigilant for acute onset manifestations and consider vitamin B12 deficiency as a differential diagnosis for the early management of pancytopenia.

## Introduction

Vitamin B12, also known as cobalamin, is a water-soluble vitamin that plays a vital role in DNA synthesis and neurologic function. Specifically, it plays an essential role in two enzymatic reactions. In the first reaction, methylmalonic acid is converted to succinyl-CoA using vitamin B12 as a cofactor. Vitamin B12 deficiency, therefore, can lead to increased serum methylmalonic acid levels. Accumulation of methylmalonyl-CoA is thought to be responsible for the neurological effects of vitamin B12 deficiency. Vitamin B12 also serves as a cofactor with folic acid in the conversion of homocysteine to methionine [1]. Methionine is necessary for purine and pyridine synthesis, the building blocks of DNA. A deficiency of vitamin B12 or folic acid may lead to increased homocysteine levels. A deficiency of vitamin B12 and the interruption of this reaction leads to the development of megaloblastic anemia [1,2].

Certain bacteria in the gastrointestinal tract of animals synthesize vitamin B12, which is then absorbed by the host animal and concentrated in its tissues. As a result, vitamin B12 is exclusively present in animal-derived foods such as liver, beef, lamb, chicken, eggs, and dairy [2]. Vitamin B12 is bound to protein and must be released before it is absorbed in humans. The breakdown process is facilitated by the acidic environment of the stomach. Parietal cells in the stomach release intrinsic factor, which binds to vitamin B12 in the duodenum. This complex of vitamin B12 and intrinsic factors helps with the absorption of vitamin B12 in the terminal ileum [1], which is then used in the above-stated two enzymatic reactions. If any of these steps are interrupted, a person may be at risk of developing a deficiency, which can result in different clinical presentations of the disease [1].

There are three main causes of vitamin B12 deficiency: nutritional deficiency, malabsorption syndromes, and other gastrointestinal issues [1]. Typically, humans have a large reserve of vitamin B12 that can last up to two to five years, even in cases of severe malabsorption. However, nutritional deficiency can still occur, especially in elderly patients who consume a limited "tea and toast" diet, alcoholics, and vegans/vegetarians. Pernicious anemia is the most common malabsorption disorder associated with vitamin B12 deficiency. It is an autoimmune disease that destroys gastric parietal cells. Without these cells, intrinsic factor production is reduced, hindering vitamin B12 absorption. Additionally, any condition that prevents gastric acid production, such as atrophic gastritis, subtotal gastrectomy, or long-term use of histamine H2-receptor blockers or proton pump inhibitors for peptic ulcer disease, can cause deficiency [1]. This is because vitamin B12 which is bound to protein in food cannot be released and cleaved. Less common causes of deficiency include Whipple's disease (a rare bacterial infection that impairs absorption), Zollinger-Ellison syndrome (a gastrinoma that causes peptic ulcer formation and diarrhea), and Crohn's disease [1,3]. Individuals who have undergone intestinal surgery, have strictures or blind loops, or are infested with tapeworms or other intestinal parasites may experience bacterial overgrowth in the small intestine. This overgrowth can compete for dietary vitamin B12 and put these individuals at risk for vitamin B12 deficiency [1]. Another rare cause of vitamin B12 deficiency is congenital transport-protein deficiencies, including but not limited to transcobalamin II deficiency [1].

Serum vitamin B12 levels are typically measured to assess a person's vitamin B12 status. To diagnose a deficiency of vitamin B12, one of two criteria must be met: a serum cobalamin level below 148 pmol/L (200 ng/L) along with signs, symptoms, and/or hematological indices of vitamin B12 deficiency or a serum cobalamin level below 148 pmol/L accompanied by elevated levels of serum homocysteine and methylmalonic acid with the acknowledgment that the patient has no renal impairment such as chronic tubulointerstitial nephritis or end-stage renal disease [3]. Inadequate vitamin B12 levels can cause hematologic and neurologic manifestations, ranging from milder symptoms such as fatigue and paresthesia to severe conditions like pancytopenia and demyelination of the corticospinal tract and dorsal columns (subacute combined systems disease). Furthermore, vitamin B12 deficiency is associated with psychiatric disorders, such as impaired memory, irritability, depression, dementia, and, in rare cases, psychosis [1,3]. Here, we present a unique case with an acute onset mix of mild and severe clinical manifestations of vitamin B12 deficiency.

## Case presentation

A 49-year-old woman with no notable medical history was transferred to our emergency department. She was evaluated in another hospital's emergency room, where she presented with severe pancytopenia (Table 1) and subsequently received 4 units of packed RBC transfusions. The patient reported experiencing intermittent weakness and fatigue for the past three weeks, accompanied by blurred vision, sudden bilateral lower extremity weakness, and legs "giving out." She denied hematochezia or melena. After being admitted to our hospital, the patient underwent a laboratory evaluation which confirmed the presence of pancytopenia (Table 1). While she was ill-appearing, no jaundice, abdominal distention, or hepatosplenomegaly were noted on her admission physical examination. Following a comprehensive clinical interview, a meticulous examination of the patient's electronic medical record yielded solely the information pertaining to the patient's homeless status. Given her profound neutropenia (Table 1) and fever, the patient was placed on neutropenic precautions, and empiric antibiotics were initiated. The differential diagnosis included acute leukemia, lymphoma with bone marrow involvement, hairy cell leukemia, autoimmune process, infectious etiology (e.g., HIV, hepatitis, cytomegalovirus (CMV)), multiple myeloma, T-cell lymphoma, nutritional deficiencies, and splenic sequestration.

**Table 1:** Laboratory data for the duration of the patient's hospital stay L: low, H: high, RBC: red blood cell: MCV: mean corpuscular volume, MCHC: mean corpuscular hemoglobin concentration, RDW: red cell distribution width, WBC: white blood cell, AST: aspartate aminotransferase: ALT: alanine transaminase, LDH: lactate dehydrogenase

Laboratory parameters	On admission at previous ED	Upon admission to our ED	At discharge	Normal range
RBC count (x 1 Mil/uL)	0.81 (L)	2.44 (L)	2.97 (L)	4.20-5.40
Hemoglobin (g/dL)	3.0 (L)	7.5 (L)	9.0 (L)	12.0-16.0
Hematocrit (%)	9.0 (L)	21.4 (L)	28.2 (L)	37.0-47.0
MCV (fL)	111.1 (H)	87.7	94.9	81.0-99.0
MCHC (g/dL)	33.3	35.0	31.9 (L)	32.2-37.0
RDW (%)	13.7	16.9 (H)	17.8 (H)	11.5-14.5
WBC count (x 1,000/uL)	1.0 (L)	0.67 (L)	3.03 (L)	4.80-10.00
Absolute neutrophils count (x 1,000/uL)	0.7 (L)	0.33 (L)	1.12 (L)	1.92-8.31
Platelet count (x 1,000/uL)	28 (L)	11 (L)	88 (L)	130-400
Alkaline phosphatase (IU/L)	38 (L)	205 (H)	181 (H)	50-136
AST (IU/L)	29	411 (H)	44 (H)	15-37
ALT (IU/L)	14	376 (H)	98 (H)	13-56
Total bilirubin (mg/dL)	2.02 (H)	11.3 (H)	1.70 (H)	0.20-1.00
LDH (IU/L)	-	2,215 (H)	810	84-246
Vitamin B12 (pg/mL)	-	126 (L)	-	211-911

**Figure 1:**
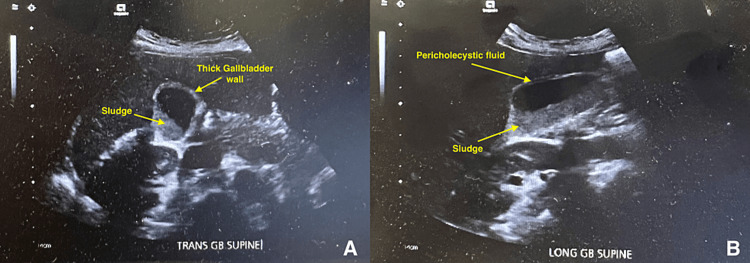
Abdominal ultrasound which shows mild gallbladder wall thickening and sludge

The hematology team closely monitored the patient and identified vitamin B12 deficiency (Table 1). The patient was started on daily cyanocobalamin 1,000 mcg via intramuscular injections. Transaminitis with hyperbilirubinemia was also noted in laboratory data (Table 1), and abdominal ultrasound showed mild gallbladder wall thickening with gallbladder sludge (Figure 1A, 1B). Computerized tomography of the abdomen and pelvis showed mild ascites and pericholecystic fluid with no gallstones or biliary ductal dilation, likely an infiltrative process. The gastrointestinal service was consulted. Broad-spectrum antibiotics were discontinued after five days as the patient remained afebrile. The patient was started on oral levofloxacin 500 mg once daily for seven days.

Hematology performed a bone marrow aspiration and biopsy, which showed a markedly hypercellular marrow with erythroid hyperplasia, dyspoiesis, and reticulin deposition, with 2.5% blasts. All other diagnostic work-ups including laboratory testing for HIV, CMV, Epstein-Barr virus (EBV), parvovirus B19, tick-borne disease, antibodies panel including Lyme serology, tuberculosis, autoimmune profile, and hepatitis panel were unremarkable. The patient's pancytopenia was deemed secondary to severe vitamin B12 deficiency per hematology. After a few days of treatment, repeat testing showed improved bone marrow recovery, with increased reticulocyte count and normalization of lactate dehydrogenase (Table 1). By hospital day 6, the patient's complete blood cell count had significantly improved (Table 1). Hematology cleared the patient for discharge and recommended that the patient continue 1,000 mcg B12 intramuscular injections daily for the next five to six days, continue folic acid supplements, and follow up with hematology outpatient in a week. The patient did not require further transfusions and remained clinically and hemodynamically stable. The patient was also recommended to follow up with her primary care physician in three to five days and advised to establish care with the gastrointestinal outpatient service.

## Discussion

Pancytopenia is a severe manifestation of vitamin B12 deficiency that results in reduced counts of all three peripheral blood cell lines [4]. The condition is identified by hemoglobin levels below 12 g/dL in women and 13 g/dL in men, platelet counts less than 150,000 per mcL, and leukocyte counts less than 4,000 per mL (or absolute neutrophil count less than 1,800 per mL) [4]. Pancytopenia typically occurs due to decreased production of cells or increased cell destruction. Autoimmune conditions (e.g., systemic lupus erythematosus, rheumatoid arthritis) and splenic sequestration from certain diseases (e.g., alcoholic liver cirrhosis, HIV, tuberculosis, malaria) can cause pancytopenia by destroying cells [4]. Aplastic anemia is pancytopenia that is caused by bone marrow failure. Etiologies include idiopathic or autoimmune factors, infections (e.g., parvovirus B19, hepatitis, HIV, CMV, EBV), drug toxicity, or chemotherapeutic agents (e.g., methotrexate, dapsone, carbimazole, carbamazepine, chloramphenicol) [4]. However, nutritional deficiencies are the main cause of pancytopenia resulting from decreased cell production [4]. Given that this patient was homeless and of low socioeconomic status, we believe that this contributed significantly to her underlying vitamin B12 deficiency.

Vitamin B12-induced pancytopenia has been described in a handful of cases in the literature. Such cases have focused on the pediatric population. In these reported pediatric cases of vitamin B12 deficiency, the patients presented with severe manifestations of the disease, including pancytopenia, hepatosplenomegaly, leukoerythroblastosis, and neurologic developmental regression secondary to known, documented nutritional deficiencies [5-7]. In similar cases within the adult population, it was concluded that a lifelong vegetarian or vegan diet rich in veggies, bread, and rice was the primary factor causing the underlying vitamin deficiency [8-10]. This is unlike our case in which past medical history, family history, and social history, even after a thorough review of the electronic medical record, were unremarkable in diagnosis. Moreover, cases in which nutritional origin could not be concluded as the attributing factor presented with chronic, severe signs and symptoms of vitamin B12 deficiency, including chronic anemia requiring blood transfusions, unintentional weight loss, and fatigue over a few months [11-13]. Our case is unique in that severe manifestations of vitamin B12 deficiency presented acutely and rapidly in the span of a couple of weeks and resolved even quicker with treatment in a matter of a couple of days.

## Conclusions

This case report highlights the importance of considering vitamin B12 deficiency as a potential cause for acute onset pancytopenia and neurological symptoms, even in patients without a clear history of risk factors. The presented case of a 49-year-old female with sudden onset of syncopal-like episodes, generalized weakness, and severe pancytopenia demonstrates the diverse clinical manifestations that can arise from vitamin B12 deficiency. While vitamin B12 deficiency is a well-known nutritional insufficiency, its varied presentations, particularly those involving acute and severe symptoms, can pose diagnostic challenges for healthcare providers. This case serves as a reminder to healthcare providers to maintain a high index of suspicion and consider vitamin B12 assessment in their diagnostic workup of patients with pancytopenia to prevent potential severe complications that can lead to higher morbidity. Moreover, the swift improvement observed with vitamin B12 supplementation highlights the importance of prompt diagnosis and comprehensive evaluation. Early recognition and treatment can prevent unnecessary interventions and hospitalization, improving patient care and healthcare cost efficiency. This case adds to the growing literature emphasizing the need for continued medical education and awareness among clinicians, ensuring that vitamin B12 deficiency is considered even in cases with unusual symptomatology.
